# CD44+ and CD133+ Non-Small Cell Lung Cancer Cells Exhibit DNA Damage Response Pathways and Dormant Polyploid Giant Cancer Cell Enrichment Relating to Their p53 Status

**DOI:** 10.3390/ijms23094922

**Published:** 2022-04-28

**Authors:** Margarita Pustovalova, Taisia Blokhina, Lina Alhaddad, Anna Chigasova, Roman Chuprov-Netochin, Alexander Veviorskiy, Gleb Filkov, Andreyan N. Osipov, Sergey Leonov

**Affiliations:** 1 School of Biological and Medical Physics, Moscow Institute of Physics and Technology, 141700 Dolgoprudny, Russia; tai2509@yandex.ru (T.B.); alhaddad.l@mipt.ru (L.A.); annagrekhova1@gmail.com (A.C.); netochin@gmail.com (R.C.-N.); filcom.gl@gmail.com (G.F.); aosipov@fmbcfmba.ru (A.N.O.); 2 State Research Center—Burnasyan Federal Medical Biophysical Center of Federal Medical Biological Agency (SRC-FMBC), 123098 Moscow, Russia; 3 N.N. Semenov Federal Research Center for Chemical Physics, Russian Academy of Sciences, 119991 Moscow, Russia; veviorskiy@gmail.com; 4 Laboratory of Medical Informatics, Novgorod Technical School, Yaroslav-the-Wise Novgorod State University, 173003 Veliky Novgorod, Russia; 5 Institute of Cell Biophysics, Russian Academy of Sciences, 142290 Pushchino, Russia

**Keywords:** non-small cell lung cancer, cancer stem cells, radioresistance, p53, γH2AX, p21, senescence-associated β-galactosidase, MITF, FAM3C, polyploid giant cancer cells

## Abstract

Cancer stem cells (CSCs) play a critical role in the initiation, progression and therapy relapse of many cancers including non-small cell lung cancer (NSCLC). Here, we aimed to address the question of whether the FACS-sorted CSC-like (CD44 + &CD133 +) vs. non-CSC (CD44−/CD133− isogenic subpopulations of p53wt A549 and p53null H1299 cells differ in terms of DNA-damage signaling and the appearance of “dormant” features, including polyploidy, which are early markers (predictors) of their sensitivity to genotoxic stress. X-ray irradiation (IR) at 5 Gy provoked significantly higher levels of the ATR-Chk1/Chk2-pathway activity in CD44−/CD133− and CD133+ subpopulations of H1299 cells compared to the respective subpopulations of A549 cells, which only excited ATR-Chk2 activation as demonstrated by the Multiplex DNA-Damage/Genotoxicity profiling. The CD44+ subpopulations did not demonstrate IR-induced activation of ATR, while significantly augmenting only Chk2 and Chk1/2 in the A549- and H1299-derived cells, respectively. Compared to the A549 cells, all the subpopulations of H1299 cells established an increased IR-induced expression of the γH2AX DNA-repair protein. The CD44−/CD133− and CD133+ subpopulations of the A549 cells revealed IR-induced activation of ATR-p53-p21 cell dormancy signaling-mediated pathway, while none of the CD44+ subpopulations of either cell line possessed any signs of such activity. Our data indicated, for the first time, the transcription factor MITF–FAM3C axis operative in p53-deficient H1299 cells, specifically their CD44+ and CD133+ populations, in response to IR, which warrants further investigation. The p21-mediated quiescence is likely the predominant surviving pathway in CD44−/CD133− and CD133+ populations of A549 cells as indicated by single-cell high-content imaging and analysis of Ki67- and EdU-coupled fluorescence after IR stress. SA-beta-galhistology revealed that cellular-stress-induced premature senescence (SIPS) likely has a significant influence on the temporary dormant state of H1299 cells. For the first time, we demonstrated polyploid giant and/or multinucleated cancer-cell (PGCC/MGCC) fractions mainly featuring the progressively augmenting Ki67^low^ phenotype in CD44+ and CD133+ A549 cells at 24–48 h after IR. In contrast, the Ki67^high^ phenotype enrichment in the same fractions of all the sorted H1299 cells suggested an increase in their cycling/heterochromatin reorganization activity after IR stress. Our results proposed that entering the “quiescence” state rather than p21-mediated SIPS may play a significant role in the survival of p53wt CSC-like NSCLC cells after IR. The results obtained are important for the selection of therapeutic schemes for the treatment of patients with NSCLC, depending on the functioning of the p53 system in tumor cells.

## 1. Introduction

Non-small cell lung cancer (NSCLC) accounts for nearly 85% of all lung-cancer cases with an estimated five-year relative survival of 8%. Radiation therapy (RT) is the standard treatment for patients with locally advanced inoperable lung cancer. However, the radioresistant cancer stem cells (CSCs) that are present inside tumors are responsible for RT failure, metastases, relapses and poor prognoses in cancer patients. Many studies indicate CD133, CD44, ABCG2 and ALDH1A1 as the most recognizable lung CSC markers [1,2], as well as the expression of common stem-related transcription factors, as octamer-binding transcription factor 4 (OCT4) and sex-determining region Y-box 2 (SOX2) are upregulated in CSCs [3,4,5,6]. The molecular mechanisms of CSC radioresistance are attributable to enhanced DNA double-strand break (DSB) repair mechanisms [7]. DNA DSBs are considered the most dangerous lesions caused by ionizing radiation (IR), leading to cell-cycle arrest, programmed cell death, chromosome aberrations and mutations [8]. H2AX phosphorylation on Ser139 (γH2AX) is a primary response to DNADSB formation and repair, and is exerted by the phosphoinositide-3-kinase-related protein kinases (PIKKs), such as ATM (ataxia telangiectasia mutated), ATR (ATM and Rad3-related), or DNA-dependent protein kinase (DNA-PK) [9,10,11]. Our previous studies suggested, that in p53null NSCLC cells, ATR phosphorylates H2AX as a result of the formation of single-stranded DNA breaks and during replication stress, such as replication-fork arrest [12,13]. ATR further activates the ATR-Chk1 pathway, thereby preventing DNA from further damage, and is thus essential to the survival of many cancers [14].

In most p53wt cancers, p21 promotes stress-induced premature senescence (SIPS) rather than apoptosis [15,16]. The most widely used marker of senescent cells is senescence-associated beta-galactosidase (SA-beta-gal), a lysosomal hydrolase [17,18]. The activity of SA-beta-gal is measured at pH 6.0, while beta-galactosidase is physiologically active at pH 4.0, which is typical of lysosomal acidic media. However, the presence of SA-beta-gal alone does not exclude the possibility of a cell being quiescent, or differentiating, as they are all progressive phenomena with intermediate transitional stages [19]. Recent studies have determined that polyploid giant cancer cells (PGCCs) emerging in tumors under the influence of hypoxia, radiation and after chemotherapy may serve as a source of CSCs [20]. The main factor leading to the formation of PGCCs is p53 deregulation [21,22]. Previously, it was thought that these cells are of no interest, as they arise from the repeated failure of mitosis/cytokinesis and enter into aging, as a result of which they lose their ability to proliferate, resulting in death over time. With the onset of favorable conditions, PGCCs undergo depolyploidization as a result of multipolar mitosis, budding or splitting with the formation of daughter cells that have the features of CSCs as well as the tumorigenicity [23]. The resulting offspring exhibit increased drug resistance and radioresistance, featuring properties of stem cells, and are capable of metastasis and relapses.

In the present study, we analyzed key DNA-damage pathways in sorted CD44−/CD133−, CD44+ and CD133+ populations of A549 (p53wt) and H1299 (p53null) cells before and after 5 Gy irradiation. We differentiated proliferation and dormant states between typical diploid and polyploid giant cancer cells (PGCCs) to show that the presence of p53wt in sorted cancerstem-like CD44+ and CD133+ populations of A549 cells leads to a p21-dependent quiescence state rather than senescence.

## 2. Materials and Methods

### 2.1. Cell Lines and Culture Conditions

Human A549 and H1299 cell lines were obtained from ATCC and cultured in RPMI-1640 medium with 10% FBS, 1% penicillin/streptomycin and L-glutamine in an incubator (humidified atmosphere, 37 °C, 5% CO_2_).

### 2.2. Cell Sorting

A549 and H1299 cells were collected by trypsinization, washed in ice-cold PBS (pH = 7.4) and 1 × 10^6^ cells per sample were incubated with Anti-CD133 Antibody, Alexa Fluor^®^ 488 conjugated (MAB4310X, Sigma-Aldrich, Darmstadt, Germany) and with monoclonal Anti-CD44−PE antibody (SAB4700187, Sigma-Aldrich, Darmstadt, Germany) for 30 min at 4 °C. After the washing steps, the labeled cells were analyzed by flow cytometry using a BD FACSMelody™ Cell Sorter (BD Life Sciences, San Jose, CA, USA). The purity of sorted populations represented 90% and was validated using Amnis ImageStreamX Mark II Imaging Flow Cytometer (Luminex Corporation, Austin, TX, USA) and analyzed using a data-analysis template created in IDEAS v6.2.

### 2.3. Irradiation

Cells were seeded in 6-well plates 24 h before irradiation with 5 Gy (dose rate of 0.85 Gy/min (2.5 mA, 1.5 mm Al filter) a 200 kV X-ray) using RUB RUST-M1 X-irradiator facility (JSC “Ruselectronics”, Moscow, Russia). The exposure was performed at room temperature.

### 2.4. Western-Blotting Analysis of OCT4 Expression

Proteins were extracted using RIPA lysis buffer and centrifuged at 14,000 g for 25 min at 4 °C, the supernatant was collected, and the total protein content was measured using the bicinchoninic-acid (BCA) assay. Proteins were separated using 8–16% SDS–polyacrylamide gel (Bio-Rad Laboratories, Mini-PROTEAN TGX Gels, Hercules, CA, USA) and transferred onto nitrocellulose membranes (7.1 × 8.5 cm, Bio-Rad Laboratories, Trans-Blot^®^ Turbo™ Trans-fer, Neuberg, Germany), which were then incubated with blocking buffer (Thermo Scientific™ Pierce™ Protein-Free Blocking Buffer, Waltham, MA, USA). The membrane was incubated with anti-OCT4 Rabbit Monoclonal antibody (dilution 1:1000; ZRB1101, Merck KGaA, Darmstadt, Germany) in blocking buffer at 4 °C overnight. The polyclonal antibody against anti-GAPDH antibody (1:1000; G9545, Sigma-Aldrich, St Louis, MO, USA) was used as a house-keeping control gene. The membrane was washed with 1× PBS (pH = 7.2–7.6), containing 0.05% Tween-20 (Pharm grade, Biolot, St. Petersburg, Russia) 3 times for 3 min each and was further incubated for 2 h with a peroxidase-conjugated secondary sheep anti-rabbit (p-SAR IgGs) (dilution 1:5000, IMTEC, Moscow, Russia) antibodies at room temperature. The formed membrane-bound immune complexes were detected using the Clarity™ Western ECL Substrate reagent Luminol/peroxide solution (dilution 1:1, Bio-Rad, Hercules, CA, USA). Blots were visualized, and the relative densities of the bands were calculated by Chem-iDoc™ MP Imaging System (170–8280) by Bio-Rad and normalized to the GAPDH control.

### 2.5. Immunofluorescence Analysis of SOX2 Expression

Cells were seeded into the 384-well plate at the density of 2000 cells/0.05 cm^2^. After 24 h, cells were fixed with 4% formaldehyde, permeabilized with 0.3% Triton X-100 and incubated in 1× PBS at pH 7.4 with 2% BSA (bovine serum albumin) for 40 min at room temperature to block non-specific antibody binding. Cells were incubated with primary rabbit anti-SOX2 antibody (dilution 1:100, AB5603, Merck KGaA, Darmstadt, Germany) for 1 h at room temperature. After 3 rinses with 1× PBS, pH 7.4, cells were incubated with secondary anti-rabbit Alexa 488 antibody (dilution 1:500; Merck KGaA, Darmstadt, Germany). Nuclei were counterstained with Hoechst (Dilution 6 μg/mL, Thermo Scientific™ Hoechst 33, 342 Solution (20 mM)). The fluorescent signal was measured at excitation/emission maxima of 496/519 using the CLARIOstar microplate reader (BMG LABTECH, Ortenberg, Germany). MARS Data Analysis Software (BMG LABTECH, Ortenberg, Germany) was used to analyze the obtained data. The data are presented as fold of change over control (RFU in the wells stained by normal rabbit IgG).

### 2.6. Spheroids Culture

A549 and H1299 spheroids were generated as free-floating spheroids cultured in Corning^®^ Costar^®^ Ultra-Low Attachment Multiple 96-well plates (Kennebunk, ME, USA). Cells were re-suspended as ten thousand cells per well and cultivated in serum-free medium (RPMI, Gibco, Life Technologies Limited, Paisley, UK), which was supplemented with 20 ng/mL epidermal growth factor (EGF Sigma-Aldrich, Darmstadt, Germany), 10 ng/mL of basic fibroblast growth factor (bFGF, Sigma-Aldrich, Darmstadt, Germany), 2 mM L-glutamine and 1% penicillin/streptomycin. Cells were cultivated for up to 10 days.

### 2.7. Immunofluorescence Analysis of FAM3C and MiTF Expression in Tumor Spheroids

Immunofluorescent staining and imaging of NSCLC tumor spheroids was performed according to protocol (https://doi.org/10.1016/j.xpro.2021.100578 accessed on 18 June 2021). Spheroids were incubated with primary rabbit polyclonal antibody to FAM3C/ILEI (dilution 1:100, Cat. # ab72182, Abcam, Cambridge, MA, USA), mouse monoclonal Anti-MiTF antibody [C5] (dilution 1:100, Cat. # ab12039, Abcam, Cambridge, MA, USA) and secondary goat anti-rabbit Alexa Fluor 488 conjugated (dilution 1:500; Cat. # A-11008, Merck Millipore, Burlington, VT, USA) and goat anti-mouse Cy5 conjugated (dilution 1:500; Cat. # AP124C, Merck Millipore, Burlington, VT, USA) antibodies. Imaging was performed using EVOS FL Auto Cell Imaging System (Thermo Fisher Scientific, Waltham, MA, USA). Microphotographs were analyzed using CellProfiler cell-image-analysis software.

### 2.8. Click-iT™ EdU Alexa Fluor 488 Proliferation Assay

Cells (at concentrations of 2000/0.056 cm^2^) were seeded in a 384-well plate for 24 h and 48 h following exposure to an extra single dose of 5 Gy. Then, EdU labeling reagent (final concentration 10 μM) was added to cell cultures and maintained in a 5% CO_2_ humidified incubator at 37 °C for 2.5 h. Then, cells were fixed in 2% (*v*/*v*) paraformaldehyde at room temperature for 15 min and incubated with 6 μg/mL Hoechst 33342 (Thermo Fisher Scientific, Waltham, MA, USA) overnight for nuclei staining at 4 °C. Following two rinses in 1× PBS, 1× EdU buffer additive was added for 1 h and incubated at room temperature protected from light. Imaging and analysis of proliferating cells were performed using the ImageXpress Micro XL High-Con-tent Screening System (Molecular Devices LLC., San Jose, CA, USA).

### 2.9. Immunofluorescence Analysis of Ki67

Cells (at concentrations of cells 2000/0.056 cm^2^) were seeded in a 384-well plate for 24 h and 48 h following exposure to an extra single dose of 5 Gy. Then, cells were washed briefly in 1× PBS (pH 7.4) and fixed with 4% formaldehyde for 15 min, followed by 3 rinses in 1× PBS (pH 7.4). After that, cells were permeabilized with 0.25% Triton X-100 for 15 min and washed then 3 times in 1× PBS (pH 7.4). After blocking cells with 6% BSA (bovine serum albumin) in 1× PBS (pH 7.4) for 1 h at room temperature, cells were incubated with mouse monoclonal Ki67 antibody (5 μg/mL, clone Ki-S5, Sigma-Aldrich, Darmstadt, Germany), and diluted in 1× PBS with 1% BSA and 0.3% TritonX-100 for 1 h at room temperature. After 3 rinses in PBS, cells were incubated for 1 h at room temperature with secondary antibody, F(ab’)2-Goat anti-Mouse IgG (H + L) Secondary Antibody, Qdot 655 with conjugate (Dilution 1:50, invitrogen), which was diluted in PBS with 1% BSA and 0.3% Triton-X 100, followed by 3 rinses in 1× PBS. Nuclei were counterstained with Hoechst (Dilution 6 μg/mL, Thermo Scientific™ Hoechst 33342 Solution). Single-cell high-content imaging and analysis of cells was performed, and the inner integrated fluorescence intensities of Ki67 staining per cell nuclei were calculated using the ImageXpress Micro XL System and MetaXpress Software (Molecular Devices LLC, San Jose, CA, USA).

### 2.10. Cell-Signaling Multiplex Assay

Cells we seeded on 6-well plates and exposed to 5 Gy of X-ray irradiation, incubated for 1 h and washed 2 times with ice-cold PBS. 1X MILLIPLEX^®^ MAP Lysis Buffer with the addition of protease inhibitors (Protease Inhibitor Cocktail Set III, Cat. No. 535140, EMD Millipore, Darmstadt, Germany) was added to each well. Cells were scraped off the dish with a cell scraper and suspensions were transferred into centrifuge tubes. Lysates were rocked for 30 min at 4 °C on a shaker and then centrifuged for 25 min at 4 °C 14,000× *g*. Protein concentration was determined using BCA assay (Thermo Scientific™ Pierce™ BCA Protein Assay Kit, Rockford, IL, USA). The MILLIPLEX^®^ MAP 7-plex DNA Damage/Genotoxicity Magnetic Bead Kit (Cat. No. 48-621MAG, EMD Millipore, St. Charles, MO, USA.) was used for the simultaneous quantification of the following 7 analytes in cell lysates: ATR (total), Chk1 (Ser345), Chk2 (Thr68), H2A.X (Ser139), MDM2 (total), p21 (total), and p53 (Ser15). The analysis was performed according to the manufacturer’s instructions. Next, 10 μg of total protein was added to each well. A lyophilized stock of cell lysate prepared from A549 cells stimulated with 5 μM camptothecin (overnight) was used as a stimulated control. The analysis was performed using Luminex MAGPIX^®^ system with xPONENT^®^ software (Luminex Corporation, Austin, TX, USA). Data were analyzed using MILLIPLEX^®^ Analyst 5.1 Software (Luminex Corporation, Austin, TX, USA). The results are represented as the MFI fold of the change in tested analytes.

### 2.11. Analysis of Senescence-Associated β-Galactosidase-Positive Cells

The proportion of senescence-associated β-galactosidase (SA-beta-gal)-positive cells was analyzed using the “Cellular Senescence Assay” commercial kit (EMD Millipore, Burlington, MA, USA, Catalog Number: KAA002). The cells were stained according to manufacturer’s protocol. The stained cells were visualized using EVOS^®^ FL Auto Imaging System (Fisher Scientific, Pittsburgh, PA, USA) with 20× objective. The proportion of SA-beta-gal-positive cells was calculated manually.

### 2.12. MTT Assay

Cells were seeded into a 96-well plate (4 × 10^3^ cells/well). After 24 h, 48 h, 72 h and 96 h, 10 µL of the MTT labeling reagent (final concentration 0.5 mg/mL) was added to each well and incubated for 2 h at 37 °C in a humidified 5% CO_2_ atmosphere. Then, 150 µL of DMSO was added to each well. Absorbance was measured at 540 nm wavelength using a CLARIO star microplate reader (BMG LABTECH, Ortenberg, Germany). Data were analyzed using MARS Data Analysis Software (BMG LABTECH, Ortenberg, Germany). Relative cell viability is represented as the percentage of metabolic activity (OD450nm) of IR-treated cells relative to the metabolic activity (OD450nm) of untreated cells (control) measured at 24 h of incubation, which was taken as 100%.

### 2.13. Statistics

Statistics were performed using the Statistica 8.0 software (StatSoft, Palo Alto, CA, USA) and GraphPad Prism 9.0.2.161 (GraphPad Software, San Diego, CA, USA) software. Statistical significance was tested using the Student t-test and Mann–Whitney U Test. The results are represented as means ± SD of three independent experiments. Significance levels were denoted by asterisks: * *p* < 0.05, ** *p* < 0.01, *** *p* < 0.001, **** *p* < 0.0001.

## 3. Results

### 3.1. Sorting Strategy of CD44+ and CD133+ Cells

A cluster of differentiation 44 (CD44), which is a non-kinase cell-surface transmembrane glycoprotein and a marker for cancer stem cells, has been reported to be associated with poor prognosis in non-small-cell lung cancer (NSCLC). However, its involvement in tumor growth has not been fully elucidated [24,25]. The expression of CD133 (Prominin-1), which is a surface glycoprotein linked to organ-specific stem cells and another marker for cancer stem cells, was described as a marker of cancer-initiating cells in different tumor types and was an independent predictor of poor prognosis of NSCLC [26,27,28]. Cells overexpressing CD44 and CD133 possess several CSC traits, such as self-renewal and epithelial–mesenchymal transition (EMT) capability, as well as resistance to radiotherapy.

**Figure 1:**
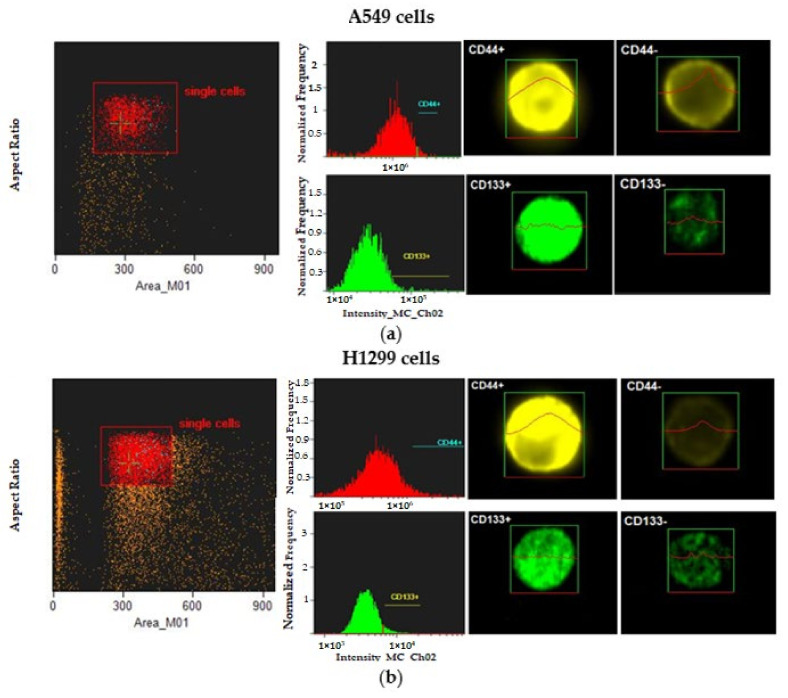
Gating strategy and representative images of CD44−, CD44+, CD133− and CD133+ populations of A549 (**a**) and H1299 (**b**) cells using Amnis ImageStreamX Mk II Imaging Flow Cytometer. The first column represents bivariate plot of BF aspect ratio versus BF area, which allows for the selection of single cells and the removal of doublets and small and large debris. Histograms of CD44 and CD133 intensity in the second column allows for the selection of CD44+ and CD133+ cells (third column) from dimly stained (CD44− and CD133−) events (fourth column).

In order to validate sorted CD44+ and CD133+ populations, cells were analyzed using Amnis ImageStreamX Mk II Imaging Flow Cytometer (Luminex Corporation, Austin, TX, USA) and IDEAS v6.2 data analysis. Bivariate plots and histograms were sequentially applied during data acquisition in INSPIRE in order to exclude the debris and ensure that the best focused single-cell images of positive and negative CD44 and CD133 cells were captured from each sample (Figure 1). The percentage of selected positive events in A549 and H1299 populations are represented in Table 1.

Thus, compared to the A549 cell line, the H1299 cell line was enriched in constitutive subpopulations of cells carrying CD44+ and CD133+ markers of CSCs.

### 3.2. Expression of Stem-Cell Transcription Markers in CD-Sorted Populations of NSCLC Cells

OCT4 and SOX2 are the key transcription factors of embryonic stem cells (ESCs). The expression of OCT4 was more frequently located at the invasive front of tumors and was significantly correlated with various aggressive behaviors of cancers, including TNM classification and the clinical stage [29]. SOX2 is a well-characterized pluripotent factor that is essential for stem-cell self-renewal, reprogramming, and homeostasis. The expression of OCT4 and SOX2 is upregulated in CSCs.

**Figure 2:**
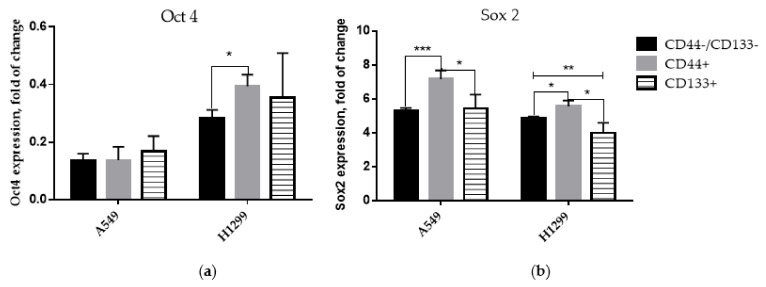
Western-blotting analysis of OCT4 (**a**) and SOX2 (**b**) expression in CD-sorted populations of A549 and H1299 cell lines. Data are means ± SD of three independent experiments. Statistical significance is denoted by asterisks, where: * *p* < 0.05, ** *p* < 0.01, *** *p* < 0.001.

The overall OCT4 expression in all the sorted populations of the H1299 cell line was higher than in the A549 cells, reaching significance in CD44−/CD133− and CD44+ cells (Figure 2a). Notably, the CD44+ subpopulation demonstrated significantly (*p* < 0.05) higher OCT4 expression compared to the CD44−/CD133− subpopulation of the H1299 cell line.

On the contrary, the overall SOX2 expression level in the H1299 cells was lower compared to the A549 populations (Figure 2b). Compared to the CD44−/CD133− and CD133+ populations, the CD44+-sorted cells demonstrated significantly (*p* < 0.001 and *p* < 0.05, respectively) higher levels of SOX2 expression in both p53wt and p53null cells. These data demonstrate that CD44+ cells contain more prominent features of CSCs in NSCLC.

### 3.3. Spheroid Formation

Another characteristic of CSCs is the 3D tumor-spheroid formation in a serum-free medium. The 3D tumor spheroids are self-organizing, free-floating structures of tumor cells with a rounded morphology and predominant intercellular interactions [30].

**Figure 3:**
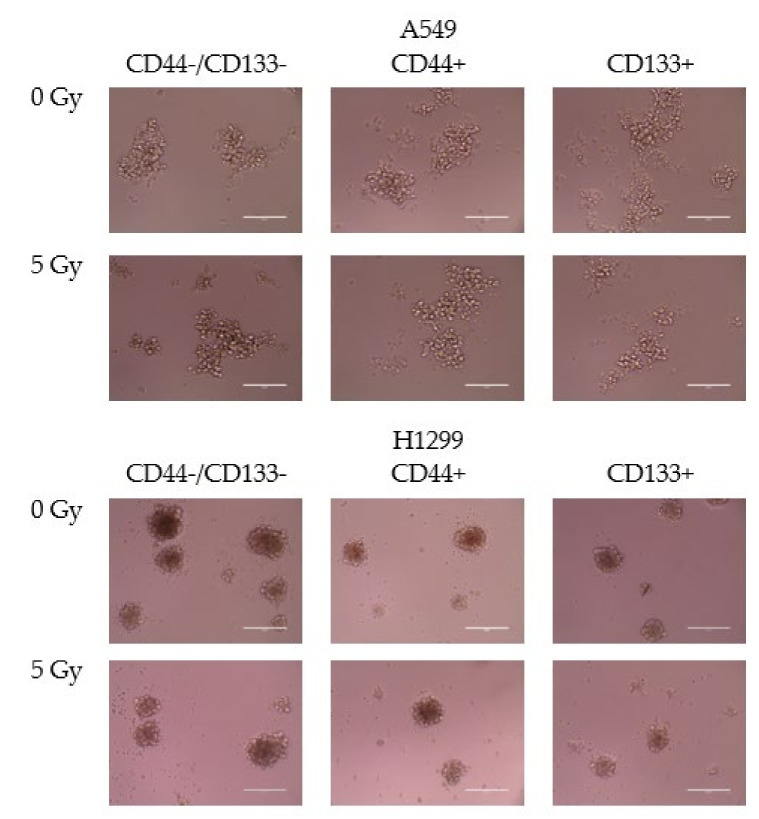
Tumor-spheroid formation in CD-sorted populations of A549 and H1299 cell lines four days after 5 Gy irradiation. Scale bar 200 µm.

Exponentially growing sorted populations of A549 (p53wt) and H1299 cells (p53null) were exposed to 5 Gy IR. Immediately after irradiation, the cells were detached from the plastic, resuspended in serum-free medium containing growth factors, and seeded at the density of 10,000 cells/well in 96-well plates. After three days, the H1299 cells formed spheroids (Figure 3). Surprisingly, although bulk A549 populations tend to aggregate, none of the CD-sorted cell populations formed spheroids. Several days later, all the H1299-derived spheroids started to shrink in size with the appearance of the high amount of cell debris in the wells (not shown). In contrast, the A549 cells sustained viable cell morphology with minimal cell debris. Thus, these results led us to speculate (or hypothesize) that under genotoxic and nutrient stress, such as IR exposure and serum starvation, functional p53 may promote entering a safer, presumably “dormant” state in order to preserve NSCLC cells from death, irrespective of their stemness.

### 3.4. Irradiation-Induced Changes in Metabolic Activity (MTT Test)

**Figure 4:**
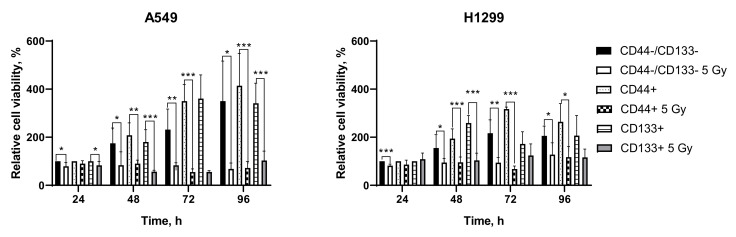
Assessment of cell viability using the metabolic MTT assay of the glycolytic NAD(P)H production at different times after IR at 5 Gy. * denotes significant differences at * *p* < 0.05; ** *p* < 0.01; *** *p* < 0.001.

To assess the relative cell viability and proliferation following 5 Gy IR exposure, we conducted the MTT assay. We observed that 5 Gy significantly diminished the rate of glycolytic NAD(P)H production of both p53wt- and p53null-derived subpopulations of NSCLC cells over 96 h following irradiation (Figure 4).

### 3.5. DNA Damage Response Pathway Profiling in CD-Sorted Populations of NSCLC Cells

The appearance of relapse after radiotherapy is associated with the presence of intratumor populations of cancer stem-like cells (CSC), which have increased resistance to genotoxic stress. It remains an open question whether CSCs really have a unique set of changes in DNA-damage response (DDR), ensuring the survival of cancer cells after IR exposure. The current observations of spheroid culture survival complement our previous data, which demonstrated that functional p53 increases clonogenic survival of X-ray-resistant sublines derived from parental NSCLC cells exposed to fractionated IR. Moreover, we also demonstrated higher numbers of γH2AX foci in H1299 cells compared to A549 cells one hour after 2 Gy exposure [13], which can be the result of inefficient DNA double-strand-break (DSB) repair or due to the formation of additional foci caused by replication stress. On the other hand, the p53null H1299 cells were more radioresistant than the H1299 subline, which expressed the functional wild-type p53 [31].

**Figure 5:**
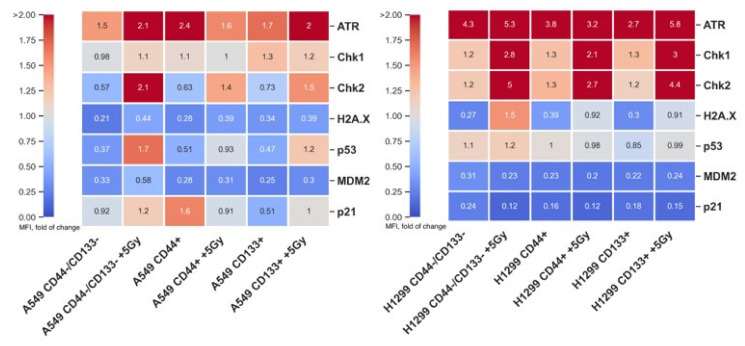
MILLIPLEX^®^ MAP 7-plex DNA Damage/Genotoxicity Magnetic Bead Panel analytes were detected in CD-sorted populations of A549 and H1299 cell lines 1 h after 5 Gy irradiation. Median Fluorescent Intensities (MFIs) are reported as fold change over the positive control (A549 cells stimulated with 5 μM camptothecin) provided by the manufacturer.

Therefore, to explore and further clarify the underlying early signaling-pathway events in response to genotoxic stress, we tested the levels of both total ATR, MDM2 and p21, and phosphorylated forms of Chk1, Chk2, H2A.X, and p53 proteins in sorted populations of A549 and H1299 cell lines one hour after 5 Gy irradiation using the MILLIPLEX^®^ MAP 7-plex DNA Damage/Genotoxicity Magnetic Bead Kit (Figure 5).

There are numerous proteins involved in the detection and repair of IR-induced DNA damage, which is initiated by the accumulation of sensor proteins such as Rad9, Rad1 and Hus1 at the site of DNA damage, and in the facilitation of checkpoint proteins’ phosphorylation conducted by the transducers ATM and ATR, which are the key kinases of the initial phase of DDR. The basal level of ATR expression in all sorted populations of non-irradiated H1299 cells was significantly (*p* < 0.05) higher than in the respective populations of A549 cells (Figure 5). The 5 Gy IR exposure further increased the levels of ATR expression over basal levels only in CD44−/CD133− and CD133+ populations of A549 and H1299 cells, whereas it decreased only in the CD44+ population of A549 cells. As expected from the ATR data, in response to IR exposure, all the sorted populations of H1299 cells demonstrated a significant (*p* < 0.05) increase in γH2AX (DNA-DSB-repair marker) levels over basal levels of the non-irradiated cells, being most prominent (almost 5,6- and 3-fold, respectively) in the CD44−/CD133− and CD133+ populations. Notably, after 5 Gy exposure, all the sorted populations of H1299 cells showed overall significantly (*p* < 0.05) higher levels of γH2AX compared to the A549 cells, which is also in agreement with our previous studies using multi-fractionated irradiation [12,13]. In contrast, the levels of γH2AX did not change in all the sorted populations of A549 cells regardless of the exposure. Together, these data may indicate that, in the absence of functional p53, the H2AX phosphorylation is likely associated with ATR activity as a result of replication-fork arrest in the presence of single-stranded DNA after IR exposure.

Checkpoint kinases, such as serine/threonine kinases Chk1 and Chk2, which are activated by ATM/ATR, are essential for cell-cycle arrest before mitosis in response to DNA damage [32]. Chk2 is stably expressed throughout the cell cycle and is mainly activated by ATM in response to DNA DSBs. In contrast, Chk1 is largely restricted to the S and G2 phases [33], and is activated in response to DNA damage or stalled replication forks. As a possible result of high ATR activity after 5 Gy exposure, all the sorted populations of H1299 cells (Figure 5) showed statistically significantly (*p* < 0.05) higher levels of Chk1 compared to both non-irradiated cell lines and the irradiated A549 cells. Chk2 showed the same pattern of expression, indicating the onset of the ATM-Chk2 pathway in the sorted populations of irradiated H1299 cells. In summary, all the sorted populations of p53null H1299 cells and p53wt A549 cells demonstrated a significant increase in IR-induced levels of ATR/ATM-Chk1/Chk2- and ATM-Chk2-pathway activity, respectively, suggesting a more prominent arrest before mitosis in CD44−/CD133− and CD133+, but not in CD44+ populations of H1299 cells.

DNA damage leads to rapid and substantial multisite phosphorylation of the DDR effector protein p53, which is initially nucleated through the phosphorylation of serine 15. The phosphorylation of p53 on Ser15 in response to DNA damage is mediated through the ATM and ATR protein kinases [34,35,36]. This phosphorylation is required for p53 to function in the physiological context of p53-responsive promoters and suggests a key and possibly universal role even for low levels of this modification in promoting p53-transcription function [37]. Curiously, the level of basal and IR-induced p53-Ser15 phosphorylation in all the sorted populations of p53null H1299 cells was almost equal to the level of the kit positive control, i.e., A549 cells stimulated with 5 μM camptothecin. Notably, these phosphorylation levels did not significantly change in response to IR in any of the sorted H1299 cell populations. In contrast, compared to non-irradiated cells, 5 Gy IR exposure of A549 cells expressing p53wt significantly augmented p53-Ser15 phosphorylation, especially in the CD44−/CD133− population, which was significantly (*p* < 0.001) higher than in the CD44+ and CD133+ populations.

Such a dramatic increase in p53-Ser15 phosphorylation in the CD44−/CD133− population of A549 cells was accompanied by a subtle increase in MDM2, which is another DDR effector and a negative regulator of p53 that counteracts the overactivation of p53 signaling. Indeed, MDM2 levels did not significantly change among the CD44+ and CD133+ populations of A549 cells after 5 Gy exposure. In the absence of functional p53, MDM2 expression in all the sorted populations of H1299 cells remained diminished irrespective of irradiation.

Similar to MDM2, the silencing of p53 signaling in the H1299 cells was further confirmed by the absence of changes in the lowest expression of p21, which is the p53 downstream transcriptional target, independently of irradiation. In contrast, all the sorted populations of non-irradiated A549 cells possessed high levels of basal p21 expression, with the CD44+ population having the most prominent levels. In response to 5 Gy exposure, the CD44+ cells displayed a statistically significant (*p* < 0.05) reduction, whereas the CD44−/CD133− and CD133+ cells demonstrated a significant increase in p21 expression compared to the respective sorted non-irradiated A549 cells. This reduction seems to likely be the consequence of decreased ATR expression followed by subtle p53-Ser15 phosphorylation signaling, only in the CD44^+^ population of A549 cells.

Collectively, the radioresistance of the CD44−/CD133− and CD133+ populations of H1299 cells was apparently related to the cell’s ability to establish the ATR/ATM-Chk1/Chk2-pathway-mediated arrest before mitosis, whereas the ATM/ATR-p53-p21 signaling-mediated cell dormancy is the anticipated consequence of IR-induced DDR in the same populations of less radioresistant A549 cells. The IR-induced DDR in the CD44+ populations of both cell lines was exceptional, i.e., these populations of A549 and H1299 cells likely underwent ATR/Chk2- and ATR/ATM-Chk1/Chk2-pathway-mediated arrest before mitosis, respectively. At the same time, none of the CD44+ populations of neither A549 nor H1299 developed cell-cycle arrest due to ATM/ATR-p53-p21 signaling-mediated cell-dormancy pathway.

### 3.6. Proliferation-Related Activity in CD-Sorted Populations of NSCLC Cells

The activation of the p53-p21 pathway caused by DNA damage leads to Cdk2 inhibition and arrest in the G1–G0 phase of the cell cycle [38]. Many studies suggest that p21 promotes SIPS rather than apoptosis in most p53wt cancers [15,16]. Hence, from our DDR-pathway-profiling data, it seems highly likely to anticipate either irradiation-induced G0/G1 arrest or SIPS in all the sorted populations of A549 and H1299 cells. Conversely, we suspected the least extent of dormant phenotypes (quiescence or SIPS) in the CD44+ populations of H1299 cells following the IR-induced DDR.

The inability of the H1299 cells to sustain extended spheroid growth cannot simply be explained by the reduction in the fraction of DNA-replicating cells in response to harsh stress conditions. Our data on cell-cycle analysis by flow cytometry with Propidium iodide staining show that both CD44+ and CD133+ A549-derived subpopulations decreased and substantially increased the proportion of cells in G1/S and in G2/M phases, respectively, at any time point after IR exposure (data not shown). Only the CD44−/CD133− subpopulation did not show any difference between the exposed and unexposed cells within 48 h after irradiation. The substantial increase in G2/M phases of the cell cycle represents the accumulation of cells that had been in earlier phases of the cell cycle at the time of IR exposure. This late G2/M accumulation suggests the enrichment of cells lacking the IR-induced S phase ATR-CHK1 checkpoint, confirming our results of Multiplex analysis.

In contrast to the A549-derived cells, the H1299-derived CD44−/CD133− subpopulation demonstrated a significant decrease and increase in cell fractions in the G1&S and G2/M phases, respectively, albeit only at 48 h after irradiation (data not shown). Both CD44+ and CD133+ H1299-derived subpopulations did not significantly change the fraction of cells in different phases of cell cycle by 48 h after IR exposure. These results might indicate that in the absence of functional p53, the 5 Gy IR exposure does not cause cell-cycle arrest in CSC-like (CD44+ &CD133+) subpopulations of H1299 cells despite significant activation of IR-induced ATR-CHK1/2-pathway signaling.

**Figure 6:**
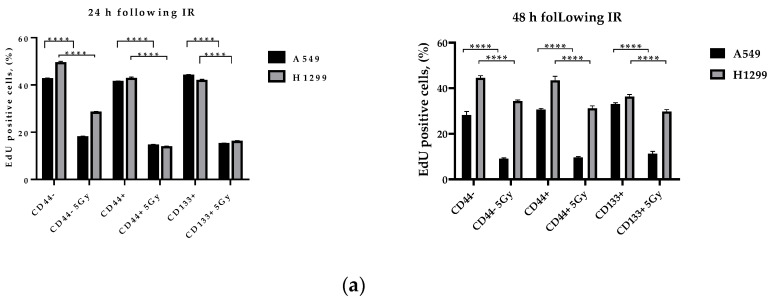

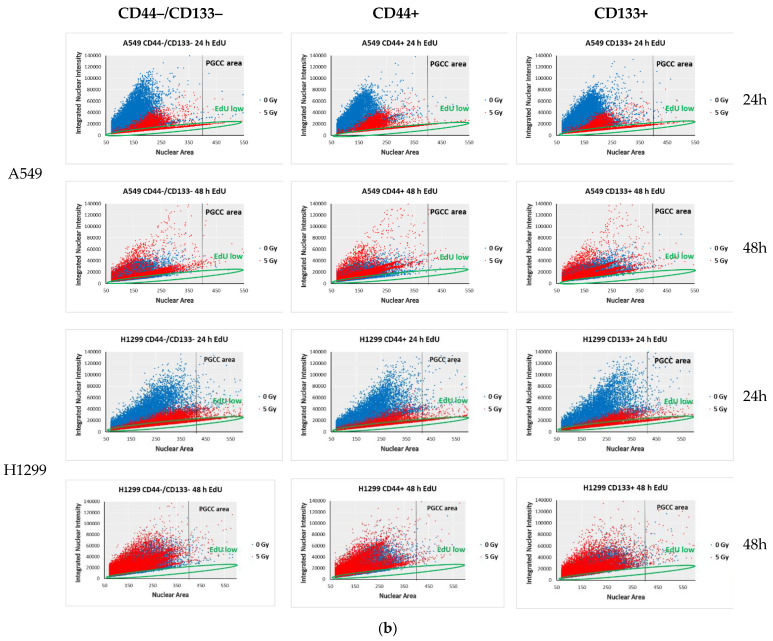
EdU-coupled fluorescence-intensity analysis of distinct cell-cycle characteristics. (**a**) The proportion of EdU-positive cells in CD-sorted populations of NSCLC cells 24–48 h after irradiation. (**b**) Using the ImageXpress Micro XL High-Content Screening System, we analyzed EdU fluorescence in PGCC 24–48 h after 0 Gy (blue dots) or 5 Gy (red dots) exposure The nuclear area of 400 nm was used as a threshold for PGCC cells according to the literature. Green circles demonstrate EdU^low^ area of “quiescent” cells. Data are means ± SD of three independent experiments. Statistical significance is denoted by asterisks, where: **** *p* < 0.0001.

Indeed, IR exposure decreased the fraction of EdU+ cells in all the sorted populations of NSCLC cells (Figure 6a). However, in the absence of functional p53, H1299-derived cells diminished the fraction of EdU+ cells to a lesser extent than the A549-derived cells, suggesting a faster entry into the DNA-replication phase of proliferation than the p53wt A549-derived cells at 48 h after irradiation (Figure 6a). These observations led us to investigate in-depth whether individual sorted populations had left the cell cycle and entered quiescence or stress-induced premature senescence (SIPS) after IR exposure.

Cell dormancy (either quiescence or SIPS) in response to genotoxic stress is often related to the formation of giant cancer cells, either multinucleated (MGCC) and/or polyploid (PGCC), in relation to their p53 status [39,40,41]. Recently, we demonstrated that p53null NSCLC cells augment polyploid giant-cancer-cell (PGCC) fractions, irrespective of IR regimen [42]. New data have demonstrated that the careful quantification of Ki67 antibody staining could reveal more than simply whether a cell is in the proliferative state: it can additionally distinguish a rapidly cycling cell with a very short quiescence from a slowly cycling cell that spends long periods in quiescence prior to re-entering the cell cycle [43]. Besides, a strict correspondence between the amounts of EdU-substituted DNA and the intensities of EdU-coupled fluorescence [44] led to our subsequent use of EdU in experiments aimed at estimating accurate cell-cycle parameters of sorted populations of NSCLC cells.

**Figure 7:**
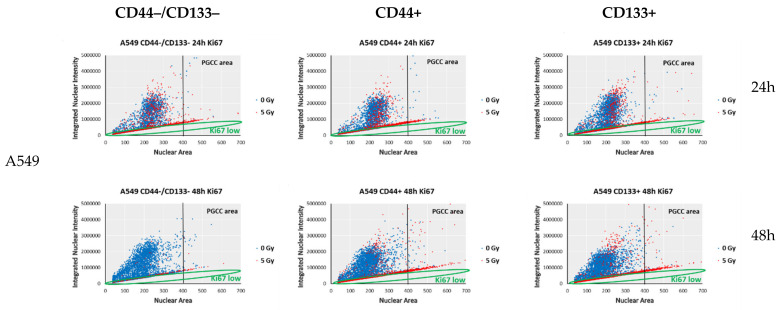

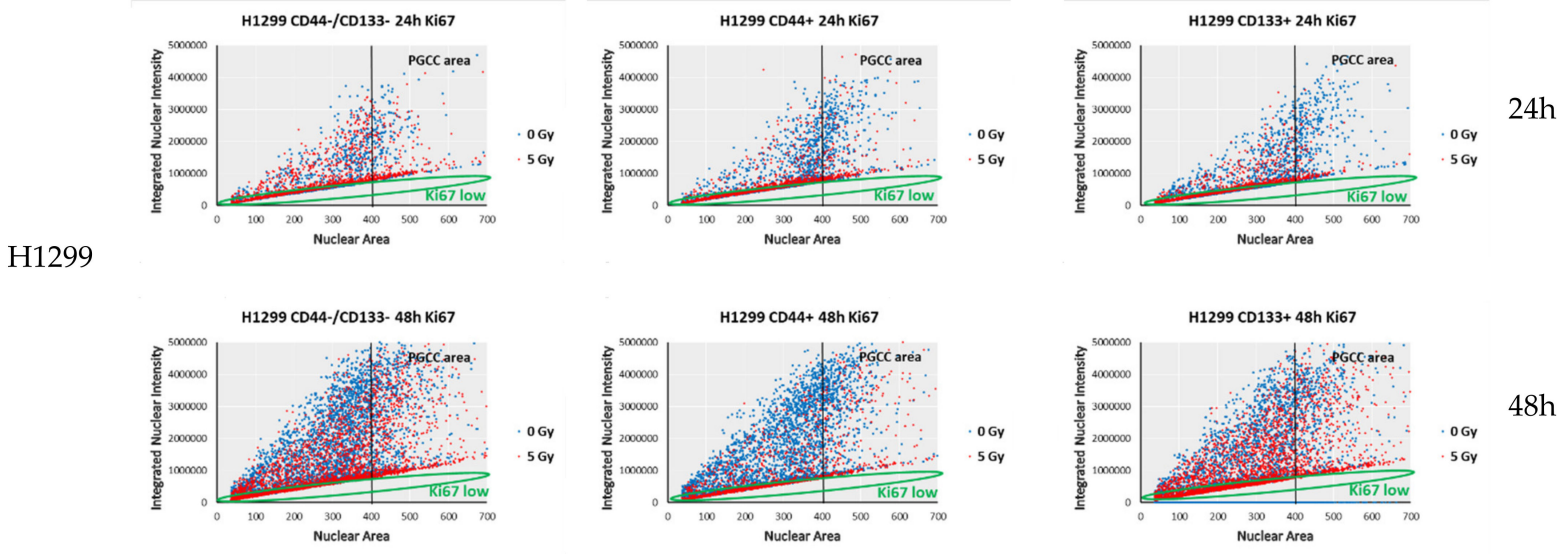
Ki67-coupled fluorescence-intensity analysis of distinct cell-cycle characteristics 24–48 h after 0 Gy (blue dots) or 5 Gy (red dots) exposure. The ImageXpress Micro XL High-Content Analysis of Ki67 fluorescence intensities in sorted populations. Green circles demonstrate Ki67^low^ area of “quiescent” (G0/G1 arrested) cells. Data are means ± SD of three independent experiments.

Hence, we took advantage of using single-cell high-content imaging and analysis to quantify the integrated intensities (IIs) of both Ki67 and EdU fluorescent signal per cell nucleus with respect to the nucleus area (reflecting either nucleus size or amount of nuclei per diploid or MGCC/PGCC cells, respectively). Plotting IIs of either Ki67 (Figure 7) or EdU (Figure 6b) staining vs. nuclear area estimated by Hoechst 33342 staining allows a relation of the nuclear area of individual cancer cells to their either cycling/heterochromatin remodeling or DNA-replication capability, respectively. Following the direct correlation of human cell ploidy and nuclear-area size [45], and the proposed characteristic of PGCCs as the tumor cells with nuclei at least three times the size of the nuclei of diploid cells [20], we first estimated the PGCC threshold (marked as “PGCC area” on Figure 6b and Figure 7) of the nuclear area of A549 and H1299 cells as> 406µm^2^, and> 420µm^2^, respectively. Such threshold values enabled MGCC/PGCC’s cycling/heterochromatin remodeling and division-capability analysis separately from the bulk of the sorted NSCLC cells.

We used EdU-coupled fluorescence-intensity analysis to characterize cell types featuring highly distinct cell-cycle characteristics. After 24 h of cultivation, every non-irradiated sorted NSCLC cell line (A549 and H1299) consisted of two populations (blue dots on Figure 6b). First, a presumably dormant slow-cycling/proliferating population (SCP), whose nuclear-area values are directly correlated with IIs of EdU limited to certain fixed and relatively low levels (approx., max value = 40,000 RFU for EdU). Another population with the scattered distribution of higher IIs of EdU-coupled fluorescence (approx., max value = 80,000 RFU for EdU) at different nuclear-area values, which likely represents a rapidly cycling/proliferating population (RCP). This distribution of all sorted populations into SCP and RCP and the ratio of background values of maximum intensities were sustained even after 48 h of the culture of non-irradiated A549 and H1299 cells. Having proved the good stoichiometric properties of incorporating EdU into newly synthesized DNA, it was demonstrated that cells in the G1 phase (DNA content = 2N; lower intensity peak) of the cell cycle contain half the amount of EdU DNA and, accordingly, emit half of the average fluorescence intensity found in cells at the S–G2 phase (DNA content = 4N; higher intensity peak) of the cell cycle [44]. Based on these data, it can be reasonably assumed that non-irradiated SCPs of both cell lines are most likely mainly in the G1–G0 phase of the cell cycle, whereas RCPs are at the S–G2 phase of the cell cycle. Of note, compared to A549 cells, all the sorted populations of non-irradiated H1299 cells contained substantially more MGCC/PGCCs (see “PGCC area” on Figure 6) belonging mainly to RCPs at any time of cultivation. Conversely, most non-irradiated MGCC/PGCCs of A549 cells apparently belonged to the SCP.

It should be noted that 24 h after irradiation, all the sorted populations of both cell lines showed a decrease in II levels of EdU-coupled fluorescence of the RCP cells to a level close to that of the non-irradiated SCP cells. In doing so, irradiated SCP cells from both cell lines appeared to exhibit further-reduced levels of EdU-coupled fluorescence with values lower than those of the non-irradiated cells, suggesting that this population was in the G1–G0 transition phase. IR augmented the formation of EdU^low^ PGCCs 24 h after exposure (Figure 6b, red dots, “PGCC area”) in all the sorted populations of both A549 and H1299 cells. At 48 h after irradiation, all the sorted populations of wild-type p53 A549 cells had significantly fewer cell counts (including MGCC/PGCCs), with rapidly cycling/proliferating cells in the G2–S (highest EdU-coupled fluorescence) phase of the cell cycle compared to the same populations of H1299 cells lacking p53. At the same time, the non-irradiated diploid fraction of A549 cells decreased the proportion of S phase cells from 1.3 to 2.8 times by 48 h (not shown).

In our study, the SCP subpopulations (both irradiated and non-irradiated) with the lowest levels of EdU–coupled (EdU^low^) fluorescence (Figure 6b) also demonstrated low IIs of Ki67-coupled fluorescence (Ki67^low^ cells) (Figure 7), resembling the features of “spontaneous G0” or the spontaneous quiescent phase of the cell cycle [46]. The spontaneous quiescent (denoted as “spG0” in Figure 6b) state differed from the classical deep quiescent G0 state in that spG0 cells did not have distinctively low levels of Ki67- and EdU-coupled fluorescence, as would be expected if Ki67 was ‘‘off’’ in deep quiescence forced by different stress conditions [43]. Indeed, deep quiescent G0-like (EdU^low^ and Ki67^low^, red dots on Figure 6b and Figure 7, respectively) SCP subpopulations clearly appeared in all the sorted populations of A549 and H1299 cells at 24 h after IR exposure.

PGCC/MGCC fractions featuring SCP were progressively augmented only in the CD44+ and CD133+ A549 cells, demonstrating the stable Ki67^low^ phenotype at 24–48 h after irradiation (Figure 7, red dots, PGCC area), which was not evident in the CD44−/CD133− population of the same cell line. In contrast, PGCC/MGCC fractions of all the sorted H1299 cells were enriched mostly in cells featuring RCP (Ki67^high^), suggesting their increase in cycling capability after 24–48 h of exposure. Interestingly, the least RCP enrichment was observed in CD44+ H1299 cells within the same period.

**Figure 8:**
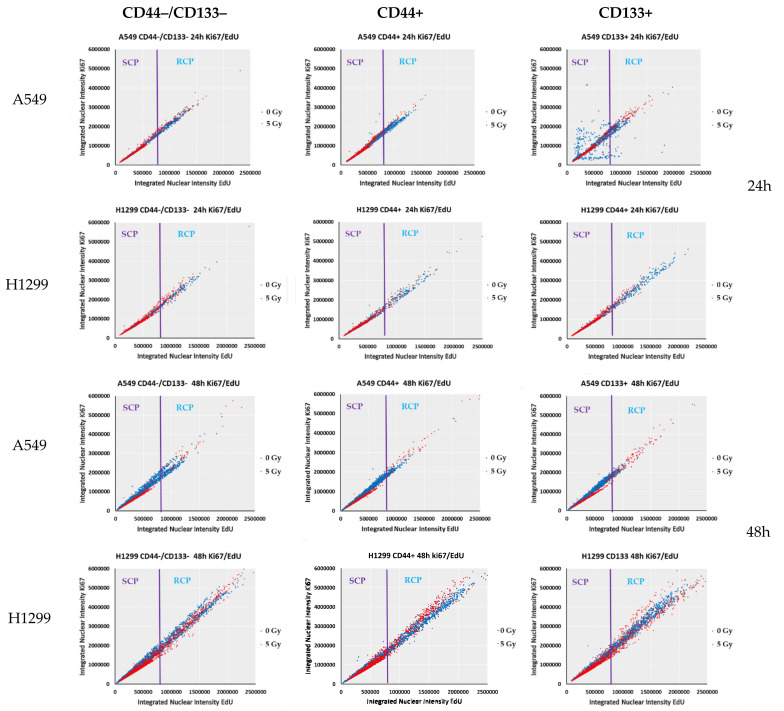
Click-IT EdU- and Ki67-coupled fluorescence measured simultaneously in sorted A549 and H1299 cells. The single-cell EdU and Ki67 quantitative fluorescence analysis is presented to differentiate between slow-cycling/proliferating population (SCP) (Ki67^low^/EdU^low^) and rapidly cycling/proliferating population (RCP) (Ki67^high^/EdU^high^) cohorts within each sorted cell population of irradiated (red dots) and non-irradiated (blue dots, control) cells at 24 h and 48h of cultivation after 5 Gy irradiation. Data are representative of three independent experiments.

To compare the cells exiting cell cycle (slow/non-cycling & proliferating, such as in SCPs) in both cell lines, we estimated the ratio of integrated intensities of EdU- and Ki67-coupled (X- and *Y*-axis, respectively on Figure 8) fluorescence that were simultaneously measured in the nuclei of the same irradiated and non-irradiated cells. Compared to non-irradiated cells, most of the populations of irradiated A549 and H1299 lines exited the cell cycle and accumulated in presumably dormant populations exhibiting extremely low EdU- and Ki67-coupled (Figure 8) fluorescence intensities in the same cells that had a limited II’s values 24 h after irradiation. A significantly smaller, presumably active cycling/proliferating population of these cells exhibited increased linearly related fluorescence intensities greater than those of the non-irradiated cells. In summary, the number of A549 cells featuring SCP (Ki67^low^/EdU^low^) and RCP (Ki67^high^/EdU^high^) subpopulations was significantly lower than the H1299 cells. These data suggest the fractions of A549 cells exiting and re-entering the cell cycle were much smaller than in the H1299 cells, the CD44+ fraction of which was likely more frequently exiting from the “dormant” state by 48 h after 5 Gy exposure.

### 3.7. The Proportion of SA-Beta-Gal Positive Cells in Response to IR

Senescent and terminally differentiated cells can also be Ki67 negative or weakly express Ki67 [47], and can mimic cells residing in the G0 state. Differing from quiescent cells, senescent cells should be identified based on their high senescence-associated β-galactosidase (SA-beta-gal) activity, the presence of p16, and the degradation of MDM2 [48]. The increased level of SA-beta-gal reactivity, as a prominent marker of high lysosomal activity and lysosomal content, was observed in both senescence and quiescence status, but was clearly higher in senescence [49]. SA-beta-gal is an enzyme that is accumulated in senescent and aging cells [50,51]. Our DDR-pathway-profiling data (Figure 5) demonstrated a subtle MDM2 increase in the CD44−/CD133− population of A549 cells only after IR.

Hence, to evaluate whether the activation of the p53-p21 pathway in A549 cells is also associated with increased SIPS constituting the impact on the irradiation-induced dormancy of all sorted populations of A549 and H1299 cells, we analyzed the proportion of senescence-associated beta-galactosidase (SA-beta-gal)-positive cells. Conversely, we suspected the least extent of the dormant SIPS phenotype in the CD44+ populations of H1299 cells following the IR-induced DDR.

**Figure 9:**
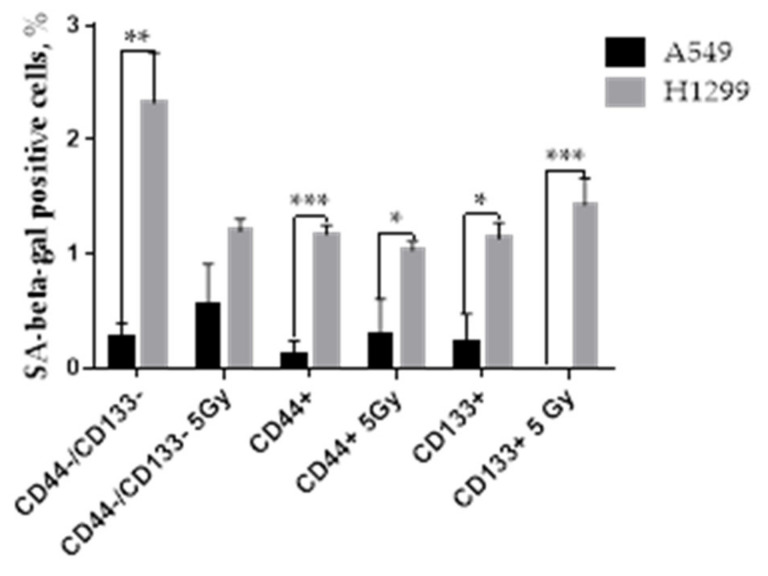
The proportion of senescence-associated beta-galactosidase (SA-beta-gal)-positive cells in CD-sorted populations of A549 and H1299 cell lines 24 h after 5 Gy irradiation. Data are means ± SEM of more than three independent experiments. Where: * *p* < 0.05, ** *p* < 0.01, *** *p* < 0.001.

Indeed, all CD-sorted populations of the non-irradiated H1299 cell line contained a significantly higher fraction of SA-beta-gal-positive cells compared to the A549 populations (Figure 9). Overall, the CD44+ populations of both p53wt A549 and p53null H1299 cells had the least basal (non-irradiated) fraction of SA-beta-gal+ cells. Exposure to 5 Gy IR significantly reduced the SA-beta-gal+ fraction of the CD44−/CD133− population of H1299 cells, without any significant changes in either the CD44+ or CD133+ population at 24 h after IR. Under the same conditions, in spite of p53-p21-MDM2 activation, the SA-beta-gal+ fraction of the CD44−/CD133− and CD44+ populations of A549 cells demonstrated a shallow increase, whereas the same fraction of the CD133+ population almost disappeared.

### 3.8. Possible Molecular Messengers of Spheroid’s Response to IR

To explore possible molecular messengers underlying the quiescence response to IR, we chose to analyze a pair of mutually connected microphthalmia-related transcription factor (MITF) and the FAM3 metabolism-regulating signaling-molecule C, also known as family with sequence similarity 3, member C (FAM3C) or a cytoplasmic interleukin-like EMT inducer (ILEI) protein.

**Figure 10:**
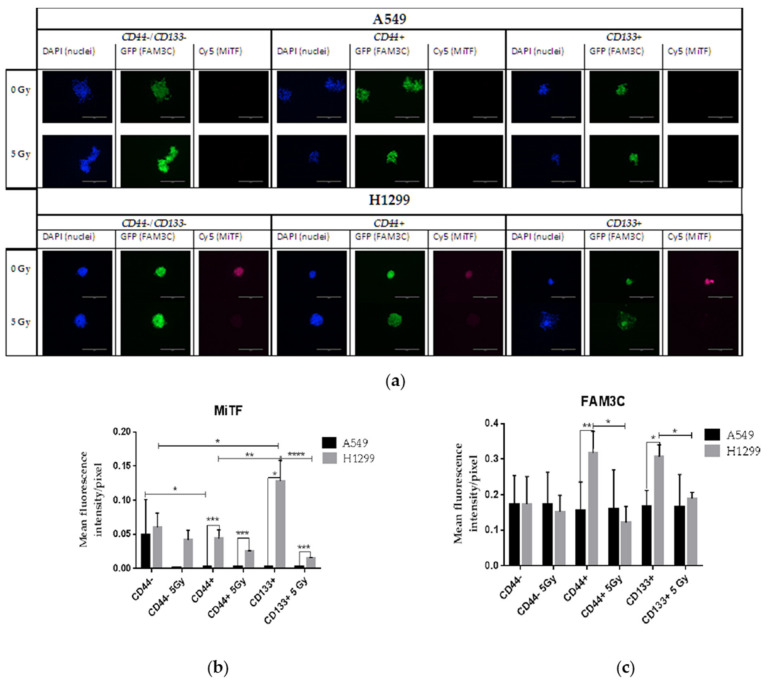
Representative microphotographs of immunofluorescently stained spheroids showing DAPI (blue), FAM3C (GFP, green) and MiTF (Cy5, pink) (**a**). The expression of MITF (**b**) and FAM3C (**c**) in spheroid cultures derived from CD-sorted populations of A549 and H1299 cell lines four days after 5 Gy irradiation. Data are means ± SEM of more than three independent experiments. Where: * *p* < 0.05, ** *p* < 0.01, *** *p* < 0.001, **** *p* < 0.0001.

Therefore, we evaluated the intracellular MITF and FAM3C expressions in spheroid cultures with and without 5 Gy IR after four days of cultivation using quantitative high-content imaging and analysis of all the sorted populations of A549 and H1299 cell lines (Figure 10).

The basal (non-irradiated) expression of MITF in the CD44+ and CD133+ populations of H1299 cells was significantly higher than in the same populations of A549 cells, which demonstrated almost negligible expression (Figure 10b). The basal MITF expression in the populations of CD44−/CD133− cells did not significantly differ between these two cell lines. In response to IR, all sorted populations of the H1299 cell line indicated a reduction in MITF expression, reaching significance in only the CD44+ and CD133+ populations of H1299 cells and in the CD44−/CD133− populations of A549-derived cells.

NSCLC cells demonstrated peculiar behavior with respect to MITF-related FAM3C expression. Indeed, compared to the A549-cell-line populations, the higher basal expression of MITF in the CD44+ and CD133+ populations of H1299 cells was well correlated with the increased cellular FAM3C expression in the same populations (Figure 10b,c). On the other hand, more than a ten-fold reduction in MITF expression in the CD133+ cells in response to 5 Gy IR downregulated FAM3C to only the basal level, which appeared to be the same in all the sorted populations of both A549 and H1299 cells, irrespective of IR (Figure 10c). These data suggest the importance of sustaining the tight control of intracellular FAM3C expression around a certain constitutive basal level in NSCLC cells. Collectively, our data indicated the MITF–FAM3C axis operative in p53-deficient H1299 cells, specifically their CD44+ and CD133+ populations, in response to IR stress.

## 4. Discussion

The ability of NSCLC cells to survive and to retain reproductive potential after radiation underlies the tumor recurrences seen in patients. The appearance of relapse after radiotherapy is associated with the presence of intratumoral populations of cancer stem-like cells (CSC), which possess increased resistance to genotoxic stress. We aimed to address the remaining open question of whether CSCs really have a unique set of pathways and effectors of DDR response that ensure the survival of NSCLC cells after IR exposure. In the present study, we analyzed key DNA-damage-response pathways and possible messengers of different fates of spheroid cultures of sorted CD44−/CD133−, CD44+ and CD133+ isogenic subpopulations of A549 (p53wt) and H1299 (p53null) cells before and after 5 Gy irradiation.

It was reported that, compared to A549 cells, the expression of CD44 and CD133, which are widely accepted CSC marker proteins, on H1299 cells was significantly lower [24] and higher [27], respectively. Nevertheless, our sorting data (Table 1) indicated that, compared to A549, the H1299 cell line was enriched in constitutive subpopulations of cells carrying CD44+ and CD133+ markers of cancer stem cells (CSCs).

In the present study, we demonstrated that in the absence of wild-type p53, CSC-like (CD44+ and CD133+) cell subpopulations might be the predominant dormant mode of escaping cell death after irradiation. As the relationship between CSCs and mutant p53 in lung adenocarcinoma is not well established, the question of whether the same role of CD44+ and CD133+ cells in escaping p53 mutant NSCLC cell death after radiotherapy remains to be addressed. In this regard, a quite recent observation indicated that triple-negative expression cases (ALDH1A1-/ CD133−/mutant p53-) in lung-adenocarcinoma patients were shown to have a much better prognosis than others [52]. This study also suggests that the detection of CSC markers and mutant p53 by immunohistochemical staining may be effective in therapeutic strategies for lung adenocarcinoma, which indirectly emphasizes the topicality of our study.

Apart from stimulating CSC formation (CD44+ or CD133+ cells), mutant or GOF p53 plays a significant role in the formation of polyploid giant cancer cells (PGCCs), which express both normal and self-renewal stem-cell genes, such as OCT4, NANOG, SOX2, along with CD44, and CD133 in ovarian and lymphoblastoid cancer cells [20,53]. In these studies, in irradiation-resistant p53-mutated lymphoma cell lines (Namalwa and WI-L2-NS, mutant p53-M237I) but not in their sensitive p53 wild-type counterparts (TK6), the low background expression of OCT4 and NANOG was upregulated by ionizing radiation, with protein accumulation evident in PGCCs. OCT4A and NANOG transcription being upregulated by irradiation in cells lacking the wild-type p53 function indicates the potential functionality of the pluripotency and self-renewal transcription network in the investigated lymphoma cell lines [53]. Here, we demonstrated that the overall basal OCT4 expression in all the sorted populations of the p53null H1299 cell line was higher than in the p53 wild-type A549 cells, reaching significance in the CD44+ over the CD44−/CD144- cells (Figure 2a). On the contrary, the overall SOX2 expression level in H1299 cells was lower compared to A549 cells (Figure 2b), with CD44+ subpopulations demonstrating significantly highest expressions in both cell lines. These data demonstrate that CD44+ subpopulations contain more prominent features of lung carcinoma CSCs. Being beyond the focus of the current study, the role and potential functionality of CSC self-renewal transcription-network proteins in p53-dependent p21-mediated NSCLC dormancy [54,55] decision after IR seems to be worthy of investigating in our forthcoming studies.

CSC radioresistance is mainly attributable to enhanced DNA-repair mechanisms [7]. Compared to the CD44−/CD133− subpopulation, CSC-like CD44+ and CD133+ H1299 cells demonstrated significantly lower levels of γH2AX, indicating more efficient repair of DNA DSBs specific to CSCs 1 h after 5 Gy IR exposure (Figure 5). However, overall γH2AX levels were higher than in every sorted subpopulation of A549 cells, which is in agreement with our previous studies [13]. We proposed that such a discrepancy is a result of additional H2AX phosphorylation caused by ATR after stalling of the replication fork. Indeed, the basal level of ATR expression in all the sorted subpopulations of non-irradiated H1299 cells was significantly (*p* < 0.05) higher than in the respective populations of A549 cells (Figure 5). The 5 Gy IR exposure further increased the levels of ATR expression over basal levels only in the CD44−/CD133− and CD133+ subpopulations of A549 and H1299 cells, whereas it decreased only in the CD44+ subpopulation of A549 cells. The increase in IR-induced levels of ATR/ATM-Chk1/Chk2- and ATM-Chk2-pathway activity in all the sorted subpopulations of p53null H1299 cells and p53wt A549 cells, respectively, suggested a more prominent arrest before mitosis in the CD44−/CD133− and CD133+, but not in the CD44+ populations of H1299 cells.

In the absence of functional p53, H1299-derived cells diminished the fraction of EdU+ cells to a lesser extent than A549-derived cells, suggesting that they entered the DNA-replication phase of proliferation faster than p53wt A549-derived cells at 48 h after irradiation (Figure 6a). In response to replication stress, replication protein A (RPA) binds to the unstable single-stranded DNA (ssDNA), and the long stretches of RPA-coated ssDNA adjacent to the double-stranded DNA (dsDNA) act as a platform to trigger the ATR/Chk1. RPA has been shown to interact with Rad51 and Rad52, and to modulate their activities and thus, promote DNA-DSB repair [56,57,58]. Chk1 and Chk2 may contribute differently to the formation of Rad51 foci depending on the type of DNA damage. Thus, Chk1 depletion leads to the loss of Rad51 localization to nuclear foci in response to replication arrest [59]. Cells lacking Chk2 also show an impairment in Rad51 nuclear localization, but only in the presence of IR-induced DNA DSBs [59]. Therefore, in the absence of functional p53, the increased ATR activity in H1299 cells after 5 Gy exposure together with the elevated levels of both Chk1 and Chk2 (Figure 5) indicate that the γH2AX phosphorylation appears not only at sites of “true” DNA DSBs, but also at sites of ssDNA caused by replication stress.

IR exposure of CD44−/CD133− and CD133+ A549-derived cells demonstrate the onset of the ATM/Chk2 pathway and significantly augment p53-Ser15 phosphorylation. Notably, basal and IR-induced p53-Ser15 phosphorylation did not change significantly in any of the sorted subpopulations of p53null H1299 cells. In contrast, compared to non-irradiated cells, 5 Gy IR exposure of A549 cells carrying p53wt significantly augmented p53-Ser15 phosphorylation, being significantly (*p* < 0.001) higher in the CD44−/CD133− population than in the CD44+ and CD133+ populations (Figure 5). However, in this regard, p53-dependent gene expression, as measured by the induction of selected p53-downstream genes, including the cell-cycle inhibitor p21, appears to be independent of increased phosphorylation of p53 [60], suggesting that Ser15 phosphorylation is possibly dispensable for p53 activity [61]. Ser15 phosphorylation is required for p53-dependent gene expression which, given the case of the p21 promoter, suggests that this residue may play a selective and promoter-context-dependent role in transactivation.

In turn, p21 inhibits the activity of cyclin-CDK2, CDK1, and CDK4/6 complexes, thus regulating the cell-cycle progression during the G1 and S phases. In addition to growth arrest, p21 can mediate cellular senescence via p53-dependent and independent pathways and suppress apoptosis, as shown in a variety of mammalian cells and tissues [62]. In response to 5 Gy exposure, CD44+ cells displayed the least (*p* < 0.05) p21 expression compared to their basal (non-irradiated) values, as well as both basal and irradiated values of the other sorted A549 cells. This p21 reduction seems likely to have been the consequence of decreased ATR expression followed by subtle p53-Ser15 phosphorylation signaling only in the CD44+ population of A549 cells. Additionally, the IR-induced DDR in the CD44+ subpopulations of A549 and H1299 cell lines was peculiar, i.e., these subpopulations likely underwent ATR/Chk2- and ATR/ATM-Chk1/Chk2-pathway-mediated arrest before mitosis, respectively. At the same time, none of these CD44+ populations of either cell line developed cell-cycle arrest due to the ATM/ATR-p53-p21 signaling-mediated cell-dormancy pathway.

The essence of a stress response is to protect cells from stress. Our observed low resistance of the CD44−/CD133− and CD133+ populations of H1299 cells to genotoxic and extended nutritional stress was apparently related to cell’s ability to establish ATR/ATM-Chk1/Chk2-pathway-mediated arrest before mitosis. Conversely, the higher resistance of the same populations of A549 cells might be the anticipated consequence of IR-induced ATM/ATR-p53-p21 signaling-augmented cell dormancy as the DDR. The activation of ATR/Chk2-pathway-mediated arrest before mitosis is likely a sufficient stress response to ensure the survival of CD44+ subpopulations of A549 cell line under the same stress conditions.

Notably, irrespective of IR, all the sorted subpopulations of p53null H1299 cells formed the spheroids in serum-free media, which is a feature previously attributed only to CSC-like cells [63,64]. In contrast, neither CD44+, nor other sorted subpopulations of p53wt A549 cells exhibited spheroids, forming only amorphous aggregate cultures under the same conditions. The observed overall reduced Ki67 expression in all the sorted subpopulations of A549 cells (Figure 7) was likely due to the fact that Ki67 knockout 4T1 cells also lose their ability to form spheroids in the absence of adhesion to a surface [65].

Intra- and extra-cellular FAM3C was found to be involved in biological processes such as signal transduction and cell–cell communication, which enhances tumorigenicity and metastasis in cancer, correlating with a shorter survival duration in patients with lung cancer [66,67]. Melanoma-specific phenotype switching in which high-MITF-expressing (MITF^high^) proliferative cells switch to a low-expressing (MITF^low^) invasive state was accompanied by low and high expression levels of ILEI (ILEI^low^ and ILEI^high^), respectively [68]. In NSCLC, MITF indicated a substantial correlation with chemoresistance of A549 cells to cisplatin [69]. In contrast to melanoma observation, compared to A549 spheroid cultures, higher basal intracellular expression of MITF in CD44+ and CD133+ spheroid cultures of H1299 cells were well correlated with their increased cellular FAM3C expression. On the other hand, more than a ten-fold reduction in the MITF expression in CD133+ cells in response to 5 Gy IR downregulated FAM3C to only the basal level, which appeared to be the same in all the sorted populations of both A549 and H1299 cells irrespective of IR. These data suggest the importance of sustaining the tight control of intracellular FAM3C expression around a certain constitutive basal level in NSCLC cells. Collectively, our data, for the first time, indicated the MITF–FAM3C axis operative in p53-deficient H1299 cells, specifically their CD44+ and CD133+ populations, in response to IR stress, which warrants further investigation.

The fate of spheroids containing sorted H1299 subpopulations was unfavorable, whereas A549-derived aggregates remained viable even after IR exposure and prolonged cultivation in serum-free media. Such H1299 cellular infirmity seems unlikely to be due to the diminished fraction of DNA-replicating cells since, compared to A549 cells, all the sorted H1299 subpopulations contained more EdU+ cells indicating faster S phase entrance by 48 h after the onset of harsh stress conditions (Figure 6a). Previously, inhibition of the ATR-CHK1 DDR pathway in expressed pancreatic ductal adenocarcinoma (PDAC) cells resulted in ERK activation, which is a key downstream protein of KRAS [70]. Together with ERK, Chk1 inhibition resulted in enhanced growth suppression and apoptosis of KRAS expressing PDAC cells. In our study, which involved both KRAS-expressing NSCLC cell lines, the ATR-Chk1 pathway was activated following 5 Gy irradiation in H1299 but not in A549 cells. This might indicate that apoptosis induction following the mutual inhibition of both ERK and Chk1 might be KRAS-independent. However, both ERK and Chk1 might be suppressed in the absence of growth factors in serum-free spheroid cell culture, suggesting that apoptosis might be the leading cause of H1299 spheroid degradation observed in our study. Indeed, we and others have demonstrated that the survival of cancer cells after IR stress relates to cell dormancy (either quiescence or SIPS), which is often associated with the formation of giant cancer cells, either multinucleated (MGCC) and/or polyploid (PGCC), in relation to their p53 status [39,40,41] and IR regimen [42]. In cancer, the rise in radiation resistance has been attributed to slow-cycling subpopulations being hardwired to stress responses in order to promote cellular survival in harsh environments [71]. Hence, we proposed that in response to genotoxic and nutritional stress, functional p53wt may promote entering a safer, presumably more favorable “dormant” (SIPS, quiescence or their combination) state, either to protect NSCLC cells (including PGCC/MGCCs) from death or to augment their fitness in IR-stress environments irrespective of their stemness.

In an attempt to prove this assumption, we used single-cell high-content imaging and analysis to quantify the integrated intensities (IIs) of both Ki67- and EdU-coupled fluorescent signals with respect to the cell nuclei area without IR, and at 24–48 h after 5 Gy IR. Plotting the Ki67- and EdU-coupled IIs vs. nuclear-area size allowed for the evaluation of all cells, including PGCC/MGCCs, regarding their cycling and DNA-replication capability, respectively. Of note, it was genetically shown that Ki67 is not required for cell proliferation in tumors, although it is required for all stages of carcinogenesis [72]. Moreover, Ki67 is an essential mediator of ectopic heterochromatin formation and links heterochromatin organization to cell proliferation. Heterochromatin reorganization caused by Ki67 downregulation does not interfere with cell-cycle progression or cell proliferation, but likely contributes to the remodeling of gene expression [65]. At 48 h after irradiation, compared to all the sorted subpopulations of p53null H1299 cells, the same populations of p53wt A549 cells had significantly fewer cells (including MGCC/PGCCs) in the G2–S (highest EdU-coupled fluorescence) phase of the cell cycle (Figure 6b, red dots, “PGCC area”). For the first time, we demonstrated that PGCC/MGCC fractions featuring Ki67^low^ phenotype were progressively augmented only in the CD44+ and CD133+ A549 cells at 24–48 h after irradiation (Figure 7, red dots, PGCC area). In contrast, PGCC/MGCC fractions of all sorted H1299 cells were enriched mostly in cells featuring Ki67^high^ phenotype, suggesting their increase in cycling/heterochromatin reorganization capability after 24–48 h of exposure (Figure 7, red dots, PGCC area). Notably, the least enrichment of Ki67^low^ phenotype cells was observed in CD44+ H1299 cells within the same period. Here, we demonstrated appearance of fractions of slow-(EdU^low^/Ki67^low^, SCP) and rapid-cycling (EdU^high^/Ki67^high^, RCP) populations in all sorted subpopulations of A549 and H1299 cells in response to IR stress (Figure 8). Our data are consistent with recent report indicating the bifurcation of Ki67 levels follows, and therefore is a consequence of, the proliferation-quiescence decision, which is consistent with Ki67 being down-regulated in all five forms of quiescence [43]. Observed linear relationship between EdU- and Ki67-coupled fluorescence in both non-irradiated and irradiated (blue and red dots, respectively on Figure 8) cells was the further confirmation of the earlier conclusion that the variability of Ki67 expression is due to its regulation through the cell cycle [47].

In all the sorted subpopulations of radioresistant H1299 cells, the fraction featuring the RCP phenotype was significantly higher than that in the respective subpopulations of A549 cells at 48 h after IR stress (Figure 8). Thus, the IR-induced reduction in the fraction of p53wt A549 cells rapidly exiting/re-entering the cell cycle and/or reorganizing heterochromatin/remodeling gene expression likely fortifies these cells against future culturing under nutrient stress. These data corroborate previous findings that dormant cancer cells escape treatment by avoiding the S phase, which is the target of chemo- and radiotherapy [73], and the ability to enter spontaneous quiescence is beneficial not only in genotoxic-stress conditions, but also in other diverse stress conditions, suggesting that quiescence actively protects cells from exogenous stress [71]. In contrast, in the absence of p53, CD44+ and CD133+ fractions of H1299 cells more frequently appeared to be exiting from the “dormant” state by 48 h after 5 Gy exposure, which likely reduced the chances of cell survival under the same subsequent stress. Thus, the reduced cancer-cell cycling, proliferation, and/or reorganizing heterochromatin/remodeling gene expression seems to be a core feature of cellular adaptation for the survival of genotoxic stress, and on a longer timescale, of changing environments.

In the present study we showed the differential expression of DDR-pathway proteins between CD44−/CD133−, CD44+ and CD133+ populations of A549 (p53wt) and H1299 (p53null) cell populations. We did not find significant differences in proliferation activity between the CD44+ and CD133+ and compared to CD44−/CD133− populations within a cell type. Thus, our data indicated that DNA-DSB efficiency does not solely depend on the expression of cell surface marker alone but rather on their p53 status.

A number of recent findings clearly support the idea that cancer-cell quiescence and senescence are associated with different forms of dormancy that lead to distinct phenotypes that are capable of driving tumor relapse, as reviewed elsewhere [74]. Of note, senescent and terminally differentiated cells are also Ki67 negative or weakly express Ki67, the established marker of cellular quiescence [47]. A clear-cut characterization of the dormancy (G0) state that is associated with the non-proliferative conditions is quite difficult. These conditions are progressive phenomena, and there is no unique event that represents a turning point indicating that a cell has left the cell cycle and entered quiescence. A prerequisite for acquiring the quiescent phenotype is to adjust the energy demand by reducing protein synthesis, Ki67 in particular. The proteins produced before slowing down the protein translation may still be present in G0-entry cells with residual Ki67 expression detected in G0 cells [75]. Our data clearly indicate that CD133+ A549 spontaneously (without IR) quiescent (SCP or Ki67^low^/EdU^low^) cells are not a homogeneous population (Figure 8, SCP area at 24 h). In addition to the main population, having both linearly connected these proliferation and cell-cycling indicators, the SCP cells consist of cells having variable Ki67 expression with constant EdU^low^, and variable EdU-coupled fluorescence under the constantly lowest Ki67 expression. These observations seem to be in line with the earlier notion that high Ki67 expression may be counterselected in cancers, which would fit with the finding that increased levels of Ki67 arrest cell proliferation [72]. The IR treatment plausibly raises the proportion of quiescent cancer cells by killing the majority of proliferating cancer cells and/or inducing the proliferating cancer cells to quiescence. Our present data favor the last assumption.

SA-beta-gal is a lysosomal enzyme that has been widely used as a senescence marker. The activity of SA-beta-gal is measured at pH 6.0, while physiologically, beta-galactosidase is active at pH 4.0, which is the typical lysosomal acid environment [76]. During physiological stress, the lysosomal pH may increase and thus activate SA-beta-gal until lysosomes successfully cope with the stress stimulus [19]. Stalled replication forks may represent an additional stress to IR-induced DNA damage in H1299 populations, leading to transitional pH increase. It was previously shown that H1299 cells overexpressing p21^waf^ acquired a senescent phenotype, thus protecting p53-deficient cells from IR-induced apoptosis [77]. However, we did not notice any statistically significant difference between H1299 subpopulations, neither in the control nor in irradiated cells, albeit all populations of the non-irradiated H1299 cell line contained a significantly higher fraction of SA-beta-gal-positive cells compared to A549 populations (Figure 9). The mechanism that triggers the onset of basal senescence in the absence of functional p53 in sorted H1299 cells needs to be further elucidated. Exposure to 5 Gy IR significantly reduced the SA-beta-gal+ fraction of CD44−/CD133− population of H1299 cells, without any significant changes in both CD44+ and CD133+ populations at 24 h after IR. Despite p53-p21-MDM2 activation (Figure 4), the SA-beta-gal+ fraction of CD44−/CD133− and Cd44+ populations of A549 cells demonstrated a shallow increase, whereas the same fraction of the CD133+ population almost disappeared. Notably, SA-beta-gal activity can be considered as senescence only after several waves (the earliest one at 24 h after insult) of increase following a genotoxic event, and a definitive senescent phenotype can be identified by evaluating the expression of Ki67, pRPS6, and SA-beta-gal activity [19]. Nevertheless, we propose p21-mediated quiescence is the predominant surviving pathway in CD44−/CD133− and CD133+ populations of A549 cells. The loss of p21 compromises quiescence in normal fibroblasts and forebrain stem cells [78,79]. The concomitant EdU and Ki67 analysis showed reduced integrated nuclear intensity of A549 cells 24 and 48 h after irradiation (Figure 8). Thus, the presence of functional p53 allows cells to enter a dormant or “quiescence” state, which favors the survival of A549 cells following irradiation and serum-free cultivation. In contrast, p53null H1299 cells enter proliferation faster compared to A549 cells and thus undergo cell death in serum-free conditions. Although the SA-beta-gal+ fraction was small at 24 h after IR, we propose that cellular SIPS rather than “quiescence” is responsible for the temporary dormant state of p53null H1299 cells, unfavorably reducing their fitness in spheroid cultures under additional nutrient stress.

While the absence of functional p53 leads to the spontaneous formation of PGCCs in H1299 cells, p53wt A549 cells demonstrate an increase in the proportion of polyploidy only after IR exposure (Figure 6b). Prolonged incubation in favorable conditions causes PGCCs to enter the cell cycle with the subsequent splitting or budding to give rise to offspring of typical ploidy [20] (Figure 6b). At the same time, the presence of proliferating PGCCs in p53null H1299 populations may indicate the onset of endoreplication, when repeating G and S phases generate polyploid cells with multiple copies of DNA content [80]. Furthermore, PGCCs can give rise to regular-sized cancer-cell progeny through asymmetric budding, which show expression of CSC markers CD44 and CD133 [20,81]. We propose that the dormant PGCC might be the source of CSCs in NSCLC cells after IR exposure.

A limit of our study and for similar findings relies on the consideration that entering quiescence or senescence is a continuous phenomenon and hence discrete conditions do not exist. Despite the increasing number of studies suggesting that tumor relapse is due to slow-cycling cells that persist after cancer treatment, further work is needed to fully demonstrate that tumor recurrence indeed relies on non-targeted quiescent cancer cells. The quiescent cells might adopt different states of dormancy, which should be further characterized by developing genetic tools allowing for their labeling and tracking in vivo during tumor recurrence, and by single-cell RNA-sequencing methodology. With additional validation, placing tumors into these more finely divided categories could inform prognosis and the selection of patient treatments, and could provide an additional readout of tumor responses to ongoing treatments.

## 5. Conclusions

We demonstrated that in the absence of functional p53 (H1299), SIPS of CD44+ and CD133+ cell subpopulations might be the predominant dormant mode of escaping cell death after irradiation, while in A549 cells, functional p53 promotes the transition of these cells into a dormancy in the form of quiescent or slow-cycling cells. In response to genotoxic stress, compared to the H1299 cell line, all the subpopulations of A549 cells formed more slowly-cycling PGCC/MGCCs followed by cell-cycle re-entry and the formation of therapy-resistant clones with increased migratory and invasive activity. The obtained results are important for the selection of therapeutic schemes for the treatment of patients with NSCLC, depending on the functioning of the p53 system in tumor cells.

## Figures and Tables

**Table 1:** The Percentage of CD44+ and CD133+ Cells in A549 and H1299 Populations.

Cell Line	CD44+ Cells, %	CD133+ Cells, %
A549	1.8	2.61
H1299	2.81	3.39

## Data Availability

The materials and data are available from the corresponding authors.
